# Circ_0019693 promotes osteogenic differentiation of bone marrow mesenchymal stem cell and enhances osteogenesis-coupled angiogenesis via regulating microRNA-942-5p-targeted purkinje cell protein 4 in the development of osteoporosis

**DOI:** 10.1080/21655979.2021.2023982

**Published:** 2022-01-14

**Authors:** Weifu He, Xiaoyan Shi, Zhenye Guo, Huan Wang, Mingming Kang, Zhi Lv

**Affiliations:** Department of Orthopaedics, The Second Hospital of Shanxi Medical University, Taiyuan City, Shanxi Province, China

**Keywords:** Circ_0019693, MiR-942-5p, PCP4, osteoporosis, osteogenic differentiation, angiogenesis

## Abstract

Circular RNA (circRNA) is a crucial regulator in multiple human diseases, including osteoporosis (OP). However, the function of numerous circRNAs remains unclear. This study aimed to explore the role and mechanism of circ_0019693 in bone marrow mesenchymal stem cell (BMSC) osteogenic differentiation and osteogenesis-coupled angiogenesis. The expression of circ_0019693, miR-942-5p and purkinje cell protein 4 (PCP4) was measured using quantitative real-time PCR (qPCR) or Western blot. Osteogenic differentiation was monitored according to the protein levels of RUNX family transcription factor 2 (RUNX2), osteopontin (OPN) and osteocalcin (OCN) by Western blot analysis, and the activity of alkaline phosphatase (ALP). Angiogenesis was evaluated by tube formation assay. The targeting relationship between miR-942-5p and circ_0019693 or PCP4 was identified using pull-down, dual-luciferase reporter, and RNA immunoprecipitation assays. Circ_0019693 was downregulated in serum samples and bone tissues from OP patients relative to normal subjects. Circ_0019693 expression was enhanced in the stages of BMSC osteogenic differentiation. Circ_0019693 overexpression enhanced the activity of ALP and the expression of RUNX2, OPN and OCN, and its overexpression also promoted angiogenesis. However, circ_0019693 knockdown played the opposite effects. MiR-942-5p was ensured to be a target of circ_0019693, and miR-942-5p enrichment reversed the effects of circ_0019693. In addition, PCP4 was a target of miR-942-5p, and miR-942-5p inhibitor-promoted BMSC osteogenic differentiation and angiogenesis were partly repressed by PCP4 knockdown. In conclusion, circ_0019693 promotes BMSC osteogenic differentiation osteogenesis-coupled angiogenesis via regulating miR-942-5p-targeted PCP4, thus blocking the development of OP.

## Introduction

Osteoporosis (OP) is a highly prevalent systemic bone disease, characterized by bone loss and deterioration of bone microstructure [1]. The prevalence of OP is increasing due to the aging of the population [2]. The progression of OP may be attributed to the imbalance between osteoblast bone formation and osteoclast bone resorption [3,4], but its pathogenesis still needs further elucidation. It is considered that bone formation and fracture are linked to moderate osteogenesis and angiogenesis that are often impaired by reduced recruitment of osteoblasts to the damaged area in osteoporosis conditions, leading to nonunion of the fracture [2,5,6]. Nowadays, the promotion of bone marrow mesenchymal stem cell (BMSC) osteogenic differentiation is regarded as a therapeutic strategy for OP [7,8]. Therefore, it is essential to uncover the molecular mechanisms of BMSC osteogenic differentiation and osteogenesis-coupled angiogenesis.

Non-coding RNAs (ncRNAs) play considerable roles in epigenetics regulation of gene expression in multiple biological processes [9]. Accumulating reports record the pivotal roles of ncRNAs in the progression of OP [10,11]. Circular RNAs (circRNAs) are a class of newly identified ncRNAs, well-known for unique loop-closed structures. Although the role of circRNA is initially underestimated, the function of circRNA in biological processes, especially disease progression, has attracted more and more attention [12]. In view of the high stability of circRNAs in tissues, cells and body liquids, circRNAs are believed to be more promising biomarkers compared to other linear RNA molecules [12,13]. The application of circRNA sequencing and bioinformatics proves that certain circRNAs are differently expressed in OP groups compared with that in non-OP groups, hinting that several circRNAs are implicated with the development of OP [14,15]. Progressive researches illustrate that circRNAs regulate BMSC osteogenic differentiation. For example, circ_0024097 contributed to the osteogenic differentiation of BMSCs via regulating its parental gene and Wnt/β-catenin pathway [16]. Whereas, the functions of multiple OP-related circRNAs on osteogenic differentiation remain unclear. A previous study performed circRNA microarray analysis and identified that circ_0019693 was one of the upregulated circRNAs in OP serum samples [14]. Given that circ_0019693 was rarely explored in any diseases, we aimed to investigate the role of circ_0019693 in the development of OP.

CircRNAs have been widely studied for their molecular sponge effect on target microRNAs (miRNAs). With the prediction of bioinformatics tools [17,18], miRNAs targeted by circ_0019693 are easily acquired. MiR-942-5p was shown to be involved in BMSC osteogenic differentiation by a recent study [19], and it was also predicted as one of the targets of circ_0019693. We speculated that circ_0019693 might regulate BMSC biological phenotypes by targeting miR-942-5p. It is canonical that miRNAs sequester gene expression via combining with gene 3ʹUTR. Likewise, it is easy to predict the target genes of miRNA by bioinformatics [20]. Purkinje cell protein 4 (PCP4) was predicted as a target of miR-942-5p, and it was declared to be downregulated in rat models of OP [21]. It should be addressed whether the involvement of PCP4 in OP was associated with circ_0019693-mediated miR-942-5p.

This study for the first time focused on circ_0019693 and hypothesized that circ_0019693 played considerable roles in BMSC osteogenic differentiation and osteogenesis-coupled angiogenesis. Besides, we confirmed the interplays between miR-942-5p and circ_0019693 or PCP4, which provided a new mechanism to explain the role of circ_0019693. Our study aimed to disclose the role and regulatory mechanism of circ_0019693 in OP, further providing the potential management strategy for OP.

## Materials and methods

### Clinical specimen collection

A total of 43 patients with OP (including 27 females and 16 males, age range: 50–78 ages) and 17 normal controls (including 11 females and 6 males, age range: 53–80 ages) from the Second Hospital of Shanxi Medical University were enrolled in this study. Normal controls suffered from external traumatic fracture, without OP symptoms. These patients had no complications or diseases related to bone metabolisms, such as nephropathy and liver disease. Besides, before treatment, none of the patients have experienced any medical therapy. Blood samples and bone tissues were collected from these subjects, with the written informed consent from all subjects. Blood samples were left standing and centrifuged to obtain serum. This study obtained the permission of the Second Hospital of Shanxi Medical University.

### Cell treatment and cell culture

Human BMSCs purchased from Cyagen (Suzhou, China) were cultured in OriCell human BMSC complete medium (No. HUXMA-90011; Cyagen) at 37°C conditions with 5% CO_2_. BMSCs were plated in 6-well plates, and BMSC differentiation was induced by supplying osteogenic differentiation medium (No. HUXMA-90021) in cell culture medium. Osteogenic differentiation medium was renewed every 3 days, and BMSCs were induced for 3 weeks for use.

### Cell transfection

Circ_0019693 overexpression vector (circ_0019693) and empty vector control (pCD5-ciR), small interference RNA targeting circ_0019693 (si-circ_0019693) and negative control (si-con) were constructed by Geneseed (Guangzhou, China). The mimic and inhibitor of miR-942-5p (miR-942-5p and in-miR-942-5p), and their matched negative controls (miR-con and in-miR-con) were purchased from Ribobio (Guangzhou, China). PCP4 siRNA (si-PCP4) and si-con were also provided by Geneseed. Lipofectamine 3000 reagent (Invitrogen, Carlsbad, CA, USA) was utilized to perform cell transfection with the guideline of protocol.

### Quantitative real-time PCR (qPCR)

Total RNA was isolated by Trizol method (Invitrogen) and quantified by NanoDrop 2000 (Thermo Fisher Scientific, Waltham, MA, USA). For cDNA synthesis, HiScript 1st Strand cDNA Synthesis Kit (Vazyme, Nanjing, China) and miRNA cDNA Synthesis Kit (Cwbio, Beijing, China) were applied for reverse transfection. Next, the UltraSYBR Mixture (Cwbio) was used to perform qPCR reaction. Here, GAPDH was used as an internal reference for circRNA and mRNA, and U6 was used as an internal reference for miRNA. Relative expression was obtained with the application of the 2^−ΔΔCt^ method. Primer sequences were shown as follows:

circ_0019693, F: 5ʹ-AGTTGCGGACAACTCCTTTG-3ʹ and R: 5ʹ-TGCCTTCTCCTCCTCTGAGT-3ʹ; nucleolar and coiled-body phosphoprotein 1 (NOLC1), F: 5ʹ-TTCCTGCGCGATAACCAACT-3ʹ and R: 5ʹ-TGGCAGACTTGAGCCAGAAG-3ʹ; miR-942-5p, F: 5ʹ-GTATGATCTTCTCTGTTTTGGC-3ʹ and R: 5ʹ-CTCAACTGGTGTCGTGGAG-3ʹ; PCP4, F: 5ʹ-TGGGTCTCAGTCCTAGTGGG-3ʹ and R: 5ʹ-TGGAGGTTTGCTATGCGTGT-3ʹ; U6, F: 5ʹ-CTCGCTTCGGCAGCACA-3ʹ and R: 5ʹ-AACGCTTCACGAATTTGCGT-3ʹ; GAPDH, F: 5ʹ-ACAGCCTCAAGATCATCAGC-3ʹ and R: 5ʹ-GGTCATGAGTCCTTCCACGAT-3ʹ.

### CircRNA subcellular location

RNA from cytoplasmic fraction or nuclear fraction was isolated using the PARTIS kit (Invitrogen). The expression of circ_0019693 in each fraction was measured by qPCR, with β-actin as a control in cytoplasm and U6 as a control in nucleus.

### CircRNA identification

CircRNA was resistant to RNase R digestion due to the lack of 3ʹ and 5ʹ tails. Total RNA was treated with RNase R (BioVision, Milpitas, CA, USA) and then used for qPCR to detect the expression of circ_0019693 and linear NOLC1.

Oligo(dT)_18_ primers (amplifying mRNAs with poly(A) tail) and random primers were purchased from Solarbio (Beijing, China) and used as reverse transcription primers. The synthesized cDNA was used for qPCR to examine the expression of circ_0019693 and linear NOLC1.

### Western blot assay

Western blot assay was carried out to examine the expression of RUNX family transcription factor 2 (RUNX2), osteopontin (OPN), osteocalcin (OCN) and PCP4 proteins. According to the previous description [22], total protein was extracted using the RIPA method (Solarbio) and then quantified by the BCA method (Solarbio). After separating by 12% SDS-PAGE, proteins were transferred onto PVDF membranes, following by blocking with 5% skim milk. Next, the membranes were cultured with the primary antibodies (Abcam, Cambridge, MA, USA), such as anti-RUNX2 (ab236639; dilution: 1/1000), anti-OPN (ab214050; dilution: 1/1000), anti-OCN (ab133612; dilution: 1/3000), anti-PCP4 (ab197377; dilution: 1/1000) and anti-β-actin (ab8227; dilution: 1/3000). Subsequently, the membranes were cultured with HRP-conjugated secondary antibody (ab205718; dilution: 1/5000). The signaling of proteins was observed using the ECL reagent (Solarbio).

### Alkaline phosphatase (ALP) staining assay

BMSCs in 96-well plates were cultured in osteogenic differentiation medium for 21 days. Then, cells were washed with PBS and fixed with 4% paraformaldehyde. Next, cells were stained using a BCIP/NBT ALP Color Development Kit (Beyotime, Shanghai, China) according to the protocol. The differentiated cells were stained with blue violet.

## Tube formation assay

According to the previous method [23], Matrigel (BD Biosciences, San Jose, CA, USA) was plated into 96-well plates for 1 h at 37°C. The supernatant of BMSCs with various transfections was collected, and HUVECs cultured in the supernatant of BMSCs were added into the Matrigel-coated 96-well plates. After incubation at 37°C for 4 h, the efficiency of tube formation was visualized under a microscope (Nikon, Tokyo, Japan).

### Bioinformatics tools

A total of 3 databases were employed in this study, including starbase (http://starbase.sysu.edu.cn/), circular RNA interactome (https://circinteractome.nia.nih.gov/), and circbank (http://www.circbank.cn/).

### RNA pull-down assay

Biotin probes of circ_0019693 (circ_0019693 probe) and miR-942-5p (bio-miR-942-5p-WT, containing wild-type miR-942-5p sequence; bio-miR-942-5p-MUT, containing mutant-type miR-942-5p sequence) were provided by Ribobio. The probes were incubated with streptavidin magnetic beads (Thermo Fisher Scientific) for 2 h. BMSCs were lysed and then cultured with probe-coated beads overnight at 4°C. RNA compounds bound to beads were eluted and isolated for qPCR analysis.

### Dual-luciferase reporter assay

Luciferase reporter plasmids of circ_0019693-WT (containing miR-942-5p binding site), circ_0019693-MUT (containing mutant miR-942-5p binding site), PCP4-3ʹUTR-WT (containing miR-942-5p binding site), and PCP4-3ʹUTR-MUT (containing mutant miR-942-5p binding site) were constructed using pmirGLO plasmid (Promega, Madison, WI, USA). BMSCs were cotransfected with circ_0019693-WT, circ_0019693-MUT, PCP4-3ʹUTR-WT or PCP4-3ʹUTR-MUT and miR-942-5p or miR-con. After culturing for 48 h, luciferase activity was monitored using a Dual-Luciferase Reporter Assay System (Promega).

### RNA immunoprecipitation (RIP) assay

RIP assay was conducted in accordance with the protocol from the Magna RIP kit (Millipore, Bedford, MA, USA). BMSCs were lysed and then incubated with magnetic beads pre-conjugated with the antibody of anti-Ago2 (Millipore) or anti-IgG (control; Millipore) to capture RNA compounds. RNA compounds bound to beads were eluted and isolated for qPCR analysis.

## Statistical analysis

Three independent biological experiments were performed to obtain the experimental data, followed by data processing using GraphPad Prism 7.0 (GraphPad, La Jolla, CA, USA). Statistical difference in different groups was analyzed using Student’s *t*-test or analysis of variance. The correlation between two groups was analyzed using Pearson’s correlation analysis. Data were finally exhibited as the mean ± standard deviation. A *P*-value less than 0.05 was defined to be statistical difference.

## Results

We reported a circRNA, circ_0019693, whose expression was aberrantly declined in OP patients, and its expression was gradually increased during BMSC osteogenic differentiation. We conducted gain- and loss-of-function assays to explore the function of circ_0019693 and monitored that circ_0019693 overexpression enhanced BMSC osteogenic differentiation and angiogenesis. We further summarized that circ_0019693 regulated miR-942-5p-targeted PCP4 to promote osteogenesis and angiogenesis, thus affecting the progression of OP.

### Circ_0019693 expression was reduced in serum and bone tissues from OP patients

**Figure 1.**
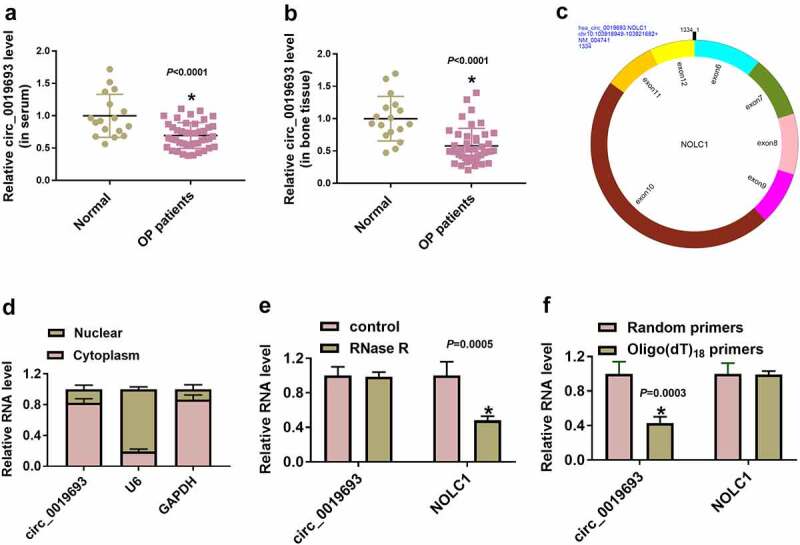
**Circ_0019693 expression was reduced in OP serum and bone tissues**. (a and b) Circ_0019693 expression was detected using qPCR in serum samples and bone tissues. (c) The formation and structure of circ_0019693. (d) The location of circ_0019693 in cytoplasm or in nucleus. (e) The stability of circ_0019693 was checked using RNase R. (f) The existence of circ_0019693 was ensured using oligo(dT)_18_ primers. *P < 0.05.

Expression analysis showed that circ_0019693 expression was strikingly decreased in serum and bone tissues from OP patients compared with that from normal subjects (Figure 1(a and b)). Circ_0019693 was derived from the exon6-exon12 of NOLC1 gene by back-splicing, with 1334 bp in length (Figure 1(c)). Subcellular location analysis presented that circ_0019693 was mainly located in the cytoplasm relative to nucleus (Figure 1(d)). After RNase R digestion, circ_0019693 expression was rarely degraded by RNase R, while NOLC1 expression was largely degraded by RNase R, verifying the existence of circ_0019693 (Figure 1(e)). In addition, circ_0019693 was hardly amplified by oligo(dT)_18_ primers, while NOLC1 could be amplified by random primers and oligo(dT)_18_ primers, which verified the circular structure of circ_0019693 (Figure 1(f)). The data indicated that circ_0019693 was upregulated in OP.

### Circ_0019693 overexpression promoted BMSC osteogenic differentiation and angiogenesis

**Figure 2.**
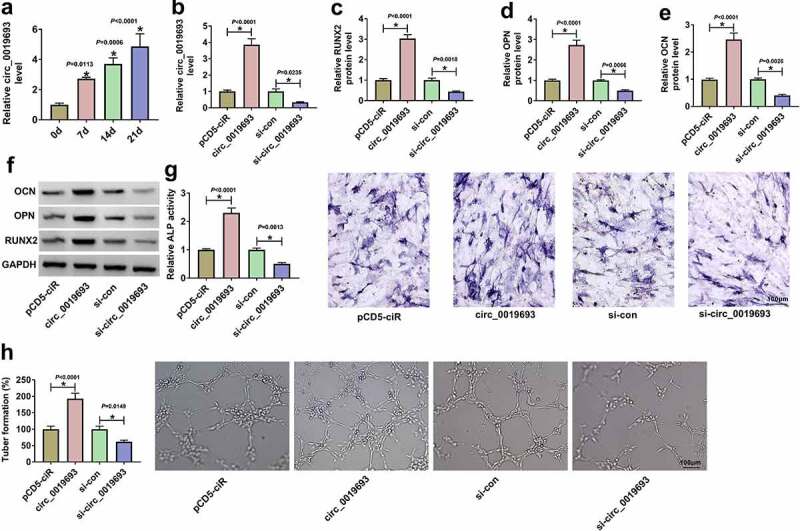
**Circ_0019693 overexpression promoted BMSC osteogenic differentiation and angiogenesis**. (a) The expression of circ_0019693 during the osteogenic differentiation of BMSCs was measured by qPCR. (b) The efficiency of circ_0019693 overexpression vector and si-circ_0019693 was checked by qPCR. (c-f) The protein levels of RUNX2, OPN and OCN affected by circ_0019693 overexpression or knockdown were measured by Western blot. (g) ALP activity was examined to determine osteogenic differentiation. (h) Cell angiogenesis was assessed by tube formation assay. *P < 0.05.

The expression of circ_0019693 was gradually upregulated during the osteogenic differentiation of BMSCs (Figure 2(a)). Gain- or loss-function assays were performed to determine the functional role of circ_0019693. The expression of circ_0019693 was markedly strengthened in BMSCs transfected with circ_0019693, while its expression was markedly reduced in BMSCs transfected with si-circ_0019693 (Figure 2(b)). Several osteogenic differentiation-related markers were quantified by Western blot. The results presented that the expression of RUNX2, OPN and OCN was notably enhanced in BMSCs with circ_0019693 overexpression but largely decreased in BMSCs with circ_0019693 knockdown (Figure 2(c-f)). Besides, ALP activity was shown to be markedly increased in BMSCs with circ_0019693 overexpression but largely decreased in BMSCs with circ_0019693 knockdown (Figure 2(g)). Tube formation assay uncovered that circ_0019693 overexpression promoted tube formation of HUVECs, while circ_0019693 knockdown blocked tube formation (Figure 2(h)). These data summarized that circ_0019693 promoted BMSC osteogenic differentiation and angiogenesis.

### Circ_0019693 targeted and negatively regulated miR-942-5p

**Figure 3.**
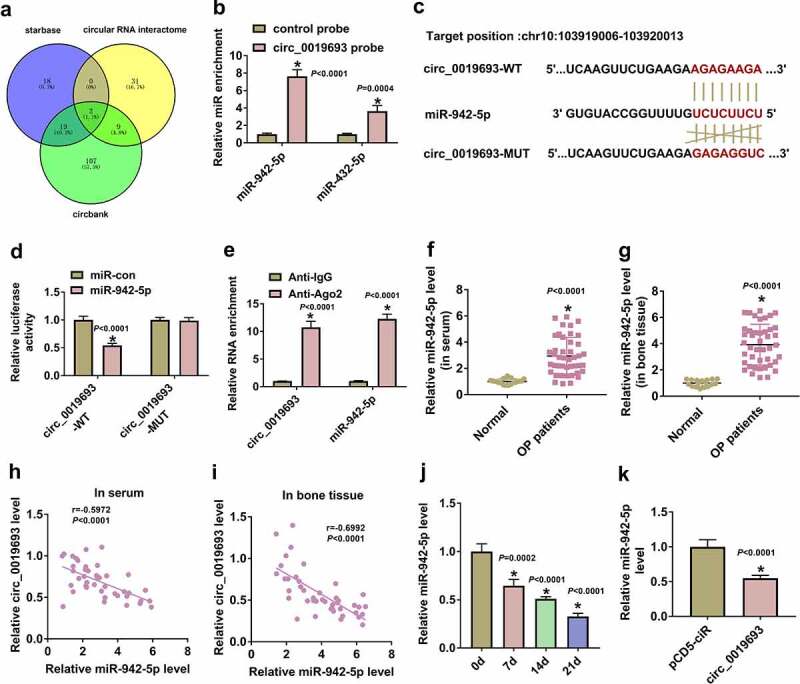
**MiR-942-5p was targeted by circ_0019693**. (a) MiRNAs potentially targeted by circ_0019693 were predicted by starbase, circular RNA interactome, and circbank. (b) Pull-down assay was performed to analyze miRNAs enriched by circ_0019693 probe. (c) The binding site between circ_0019693 and miR-942-5p. (d and e) The binding between circ_0019693 and miR-942-5p was verified by dual-luciferase reporter assay and RIP assay. (f and g) MiR-942-5p expression in serum and bone tissues was measured by qPCR. (h and i) The correlation between circ_0019693 expression and miR-942-5p expression in serum samples and bone tissues. (j) The expression of miR-942-5p during the osteogenic differentiation of BMSCs was measured by qPCR. (k) MiR-942-5p expression in BMSCs with circ_0019693 overexpression was measured by qPCR. *P < 0.05.

Starbase, circlar RNA interactome and circbank were utilized to predicted miRNAs targeted by circ_0019693, and miR-942-5p and miR-432-5p were overlapped by three databases (Figure 3(a)). We used circ_0019693 probe by pull-down assay to determine whether miR-942-5p and miR-432-5p could be enriched by circ_0019693. The data showed that miR-942-5p, compared to miR-432-5p, with more abundance, was enriched by circ_0019693 probe (Figure 3(b)). Then, we designed the mutant sequence fragment of circ_0019693 and constructed circ_0019693-WT and circ_0019693-MUT luciferase reporter plasmids (Figure 3(c)). The data revealed that miR-942-5p overexpression significantly reduced luciferase activity in BMSCs transfected with circ_0019693-WT (Figure 3(d)). In addition, circ_0019693 and miR-942-5p were both enriched in the anti-Ago2-mediated RIP group relative to anti-IgG (Figure 3(e)). The expression of miR-942-5p was notably higher in serum and bone tissues from OP patients compared with that from normal subjects (Figure 3(f-g)). Circ_0019693 expression in serum samples and bone tissues showed negative correlation with miR-942-5p expression (Figure 3(h-i)). Moreover, miR-942-5p expression was gradually declined during the osteogenic differentiation of BMSCs (Figure 3(j)). MiR-942-5p expression was markedly impaired by circ_0019693 overexpression in BMSCs (Figure 3(k)). Overall, miR-942-5p was a target of circ_0019693, and circ_0019693 negatively modulated miR-942-5p expression.

### MiR-942-5p upregulation reversed the role of circ_0019693 overexpression

**Figure 4.**
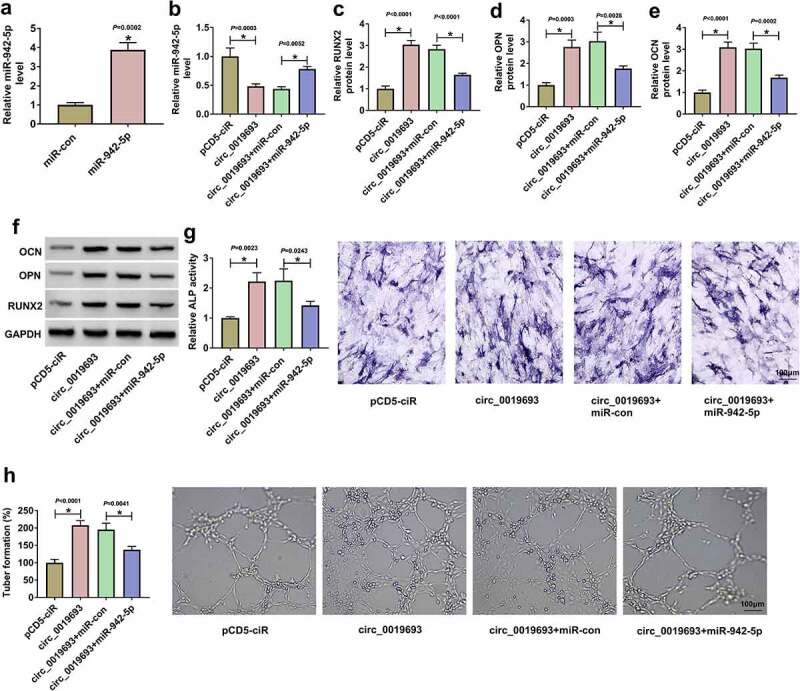
**Circ_0019693 played functions by targeting miR-942-5p**. (a) The efficiency of miR-942-5p mimic was checked by qPCR. (b) The expression of miR-942-5p in BMSCs transfected with circ_0019693 or circ_0019693+ miR-942-5p was checked by qPCR. (c-f) The protein levels of RUNX2, OPN and OCN in these cells were detected by Western blot. (g) ALP activity was measured to assess osteogenic differentiation of BMSCs in different groups. (h) Angiogenesis was evaluated by tube formation assay. *P < 0.05.

Rescue experiments were performed to determine whether circ_0019693 promoted BMSC osteogenic differentiation and phenotype changes by targeting miR-942-5p. MiR-942-5p mimic significantly enhanced the expression of miR-942-5p in BMSCs (Figure 4(a)). Then, miR-942-5p expression decreased in circ_0019693-transfected BMSCs but partially enhanced in circ_0019693+ miR-942-5p-transfected BMSCs (Figure 4(b)). The protein levels of RUNX2, OPN and OCN were strengthened by circ_0019693 overexpression, while further miR-942-5p upregulation repressed their expression levels (Figure 4(c-f)). The increased ALP activity caused by circ_0019693 overexpression was also reversed by the reintroduction of miR-942-5p (Figure 4(g)). Moreover, we found that the overexpression of circ_0019693 and miR-942-5p markedly reversed the enhanced angiogenesis of BMSCs relative to alone circ_0019693 upregulation (Figure 4(h)). These data proved that circ_0019693 overexpression promoted BMSC osteogenic differentiation and angiogenesis via targeting miR-942-5p.

### MiR-942-5p bound to PCP4 and negatively regulated PCP4 expression

**Figure 5.**
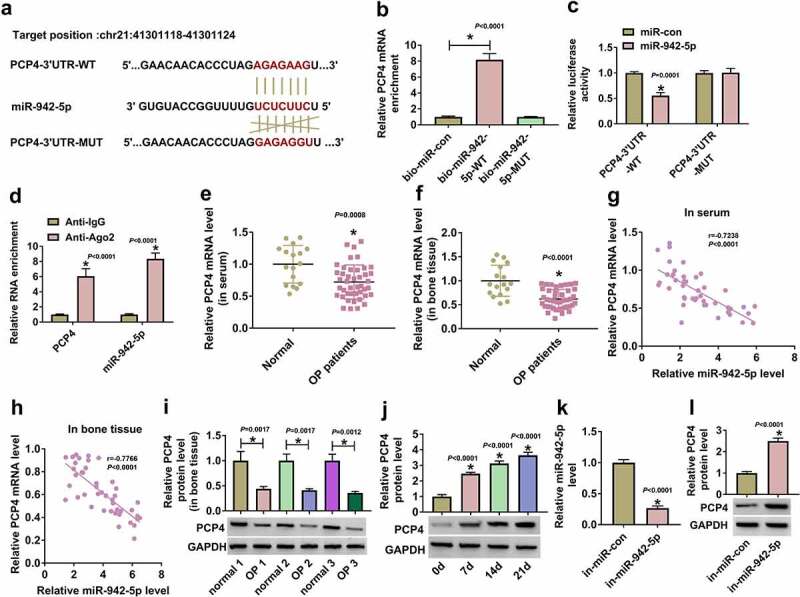
**PCP4 was a target of miR-942-5p**. (a) The binding site between miR-942-5p and PCP4 3ʹUTR was provided by starbase. (b-d) The relationship between PCP4 and miR-942-5p was validated by pull-down, dual-luciferase reporter and RIP assays. (e and f) The expression of PCP4 mRNA in serum samples and bone tissues was measured by qPCR. (g and h) The correlation between PCP4 expression and miR-942-5p expression in serum samples and bone tissues. (i) The expression of PCP4 protein in bone tissues was measured by Western blot. (j) The expression of PCP4 protein during the osteogenic differentiation of BMSCs was checked by Western blot. (k) The efficiency of in-miR-942-5p was checked by qPCR. (l) The expression of PCP4 protein in BMSCs with miR-942-5p inhibition was measured by Western blot. *P < 0.05.

The potential binding relationship between miR-942-5p and PCP4 was predicted by starbase, and then PCP4-3ʹUTR-WT and PCP4-3ʹUTR-MUT reporter plasmids were constructed (Figure 5(a)). The data from pull-down assay exhibited that high abundance of PCP4 was enriched by bio-miR-942-5p-WT probe (Figure 5(b)). Luciferase reporter assay pointed out that miR-942-5p enrichment markedly weakened luciferase activity in BMSCs transfected with PCP4-3ʹUTR-WT (Figure 5(c)). RIP assay presented that PCP4 and miR-942-5p were both enriched in the anti-Ago2-mediated RIP group relative to anti-IgG (Figure 5(d)). The expression of PCP4 mRNA was strikingly decreased in serum samples and bone tissues from OP patients compared with that from normal subjects (Figure 5(e-f)). In addition, PCP4 mRNA expression was negatively correlated with miR-942-5p expression in serum samples and bone tissues (Figure 5(g-h)). The expression of PCP4 protein in bone tissues from OP patients was also markedly declined (Figure 5(i)). However, PCP4 expression was gradually increased during the osteogenic differentiation of BMSCs (Figure 5(j)). The transfection of in-miR-942-5p markedly reduced the expression of miR-942-5p but strengthened the expression of PCP4 in BMSCs (Figure 5(k-l)). The data indicated that miR-942-5p negatively regulated the expression of PCP4.

### MiR-942-5p repression promoted BMSC osteogenic differentiation and angiogenesis by increasing PCP4 expression

**Figure 6.**
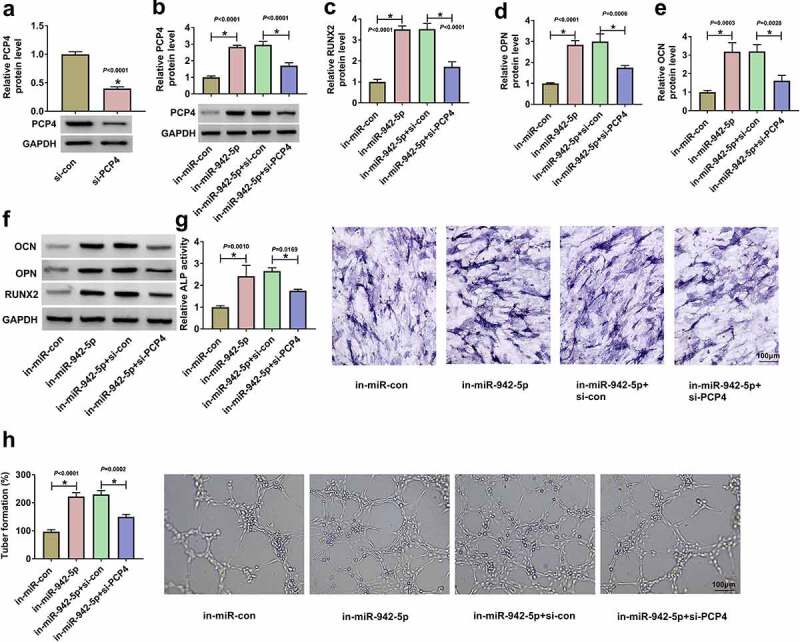
**MiR-942-5p played functions by targeting PCP4**. (a) The efficiency of si-PCP4 was checked by Western blot. (b) The expression of PCP4 protein in BMSCs transfected with in-miR-942-5p or in-miR-942-5p+si-PCP4 was checked by Western blot. (c-f) The protein levels of RUNX2, OPN and OCN in these cells were detected by qPCR. (g) ALP activity was measured to assess osteogenic differentiation of BMSCs in different groups. (h) Angiogenesis was evaluated by tube formation assay. *P < 0.05.

The expression of PCP4 was remarkably weakened in BMSCs transfected with si-PCP4 (Figure 6(a)). In addition, PCP4 expression was markedly upregulated in BMSCs transfected with in-miR-942-5p but partly repressed in BMSCs transfected with in-miR-942-5p+si-PCP4 (Figure 6(b)). The protein levels of RUNX2, OPN and OCN were enhanced by miR-942-5p suppression, while further PCP4 downregulation repressed the protein levels of these markers (Figure 6(c-f)). The strengthened ALP activity caused by miR-942-5p inhibition was partially restrained by PCP4 knockdown (Figure 6(g)). Additionally, the simultaneous knockdown of miR-942-5p and PCP4 reversed the capacities of angiogenesis promoted by alone miR-942-5p knockdown (Figure 6(h)). These findings exposed that miR-942-5p functioned in osteoporosis by targeting PCP4.

### Circ_0019693 positively regulated PCP4 expression by decoying miR-942-5p

**Figure 7.**
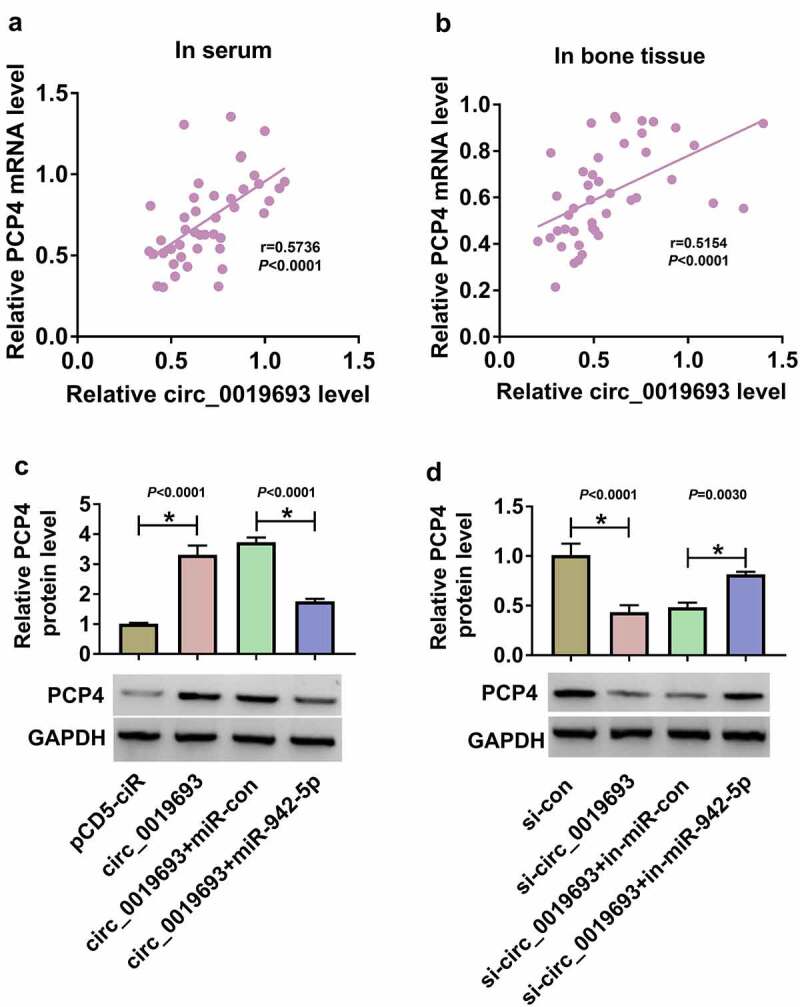
**Circ_0019693 positively regulated PCP4 expression by targeting miR-942-5p**. (a and b) The correlation between PCP4 mRNA expression and circ_0019693 expression in serum samples and bone tissues. (c) The expression of PCP4 protein in BMSCs transfected with circ_0019693 or circ_0019693+ miR-942-5p was detected by Western blot. (d) The expression of PCP4 protein in BMSCs transfected with si-circ_0019693 or si-circ_0019693+ in-miR-942-5p was detected by Western blot. *P < 0.05.

In serum samples and bone tissues from OP patients, we found that PCP4 mRNA expression was markedly positively correlated with circ_0019693 expression (Figure 7(a-b)). In addition, the expression of PCP4 protein was notably enhanced in BMSCs transfected with circ_0019693 but partly impaired in BMSCs transfected with circ_0019693+ miR-942-5p (Figure 7(c)). Unsurprisingly, the expression of PCP4 protein was significantly downregulated in BMSCs transfected with si-circ_0019693 but partially restored in BMSCs transfected with si-circ_0019693+ in-miR-942-5p (Figure 7(d)). The data suggested that circ_0019693 regulated PCP4 expression by targeting miR-942-5p.

## Discussion

Patients with OP often suffer from the delayed union of bone fractures because of impaired osteogenesis and inhibitory angiogenesis [24]. Therefore, it is necessary to exploit the factors involving in osteogenesis and angiogenesis to supply promising therapeutic strategies for OP. We mainly reported the function circ_0019693 on BMSC osteogenic differentiation and angiogenesis, thus determining the underlying role of circ_0019693 in the development of OP.

BMSCs are a kind of multifunctional, undifferentiated cells with regenerative capacity. When bone is injured, BMSCs spontaneously migrate to the bone lesion and differentiate into osteoblasts, and osteoblasts next secrete extracellular matrix (ECM) to achieve bone repair [25,26]. Blood vessels are essential to transfer oxygen, materials and nutrients, which is crucial for bone mass maintenance and bone regeneration [2,25]. Bone loss repair is a complex process with complex signaling cascades, involving osteogenic differentiation of BMSCs and well-organized angiogenesis [26,27]. Therefore, the induction of BSMC osteogenic differentiation and the coupling of osteogenesis and angiogenesis are of great significance for the repair of OP-induced bone injury. Vascular endothelial growth factor (VEGF) was required for the coupling of bone formation and angiogenesis during bone development [28]. Emerging evidence revealed that certain circRNAs were implicated with these processes. For instance, circ_0113689 was highly expressed in BMSCs during osteogenic differentiation, and its overexpression accelerated BMSC proliferation and osteogenic differentiation [29]. Besides, circ_0006215 promoted BMSC osteogenic differentiation, and the medium of BMSCs overexpressing circ_0006245 promoted HUVEC angiogenesis [19]. Consistent with these studies, we discovered that circ0019693 expression was markedly increased during BMSC osteogenic differentiation, and circ_0019693 upregulation enhanced the expression of RUNX2, OPN and OCN, and the activity of ALP. RUNX2, OPN and OCN are common markers of osteogenic differentiation [30], and ALP, produced by osteogenic cells (such as osteoblasts), is one of the most reliable markers for osteogenic differentiation [31]. The changes in the expression of RUNX2, OPN and OCN and the activity of ALP in BMSCs overexpressing circ_0019693 indicated that circ_0019693 promoted BMSC osteogenic differentiation. Besides, the supernatant of BMSCs overexpressing circ_0019693 strengthened HUVEC angiogenesis. Unsurprisingly, the knockdown of circ_0019693 showed the opposite effects.

The results from pull-down, dual-luciferase reporter and RIP assays verified that miR-942-5p was targeted by circ_0019693. Previous study reported that miR-942-5p acted as a target of circ_0006215, and its inhibition recovered the ability of BMSC osteogenic differentiation [19]. Coincidentally, circ_0074834 overexpression promoted BMSC osteogenesis and angiogenesis by targeting miR-942-5p [32]. Largely in agreement with these data, our data defined miR-942-5p as a target of circ_0019693. In function, miR-942-5p enrichment partly depleted the expression of RUNX2, OPN and OCN, the activity of ALP, and HUVEC angiogenesis in BMSCs overexpressing circ_0019693, while miR-942-5p deficiency enhanced the expression of these markers and ALP activity. The data strongly supported that circ_0019693 promoted BMSC osteogenic differentiation and angiogenesis by suppressing miR-942-5p.

To further exploit the functional mechanism of circ_0019693, the downstream mRNA molecules of miR-942-5p were screened. PCP4 attracted our attention because its expression was shown to present an upward trend during BMSC osteogenic differentiation [33]. Bioinformatics study exposed that PCP4 was associated with calcium deposition and the regulation of CaM-dependent protein kinase, hinting that osteogenic differentiation was enhanced by PCP4 depending on PCP4-induced calcium deposition [33]. In addition, PCP4 expression was declined in rat models of OP, and PCP4 overexpression promoted the differentiation of osteoblasts obtained from OP rat models [21]. Consistently, our data presented that PCP4 expression was notably decreased in OP patients but gradually enhanced during osteogenic differentiation in BMSCs. PCP4 knockdown reversed the role of miR-942-5p inhibition, leading to the inhibition of BMSC osteogenic differentiation and angiogenesis.

## Conclusion

Taken together, the expression of circ_0019693 was declined in serum samples and bone tissues from OP patients, and its expression was gradually increased in BMSCs during osteogenic differentiation. Circ_0019693 overexpression promoted BMSC osteogenic differentiation and enhanced osteogenesis-coupled angiogenesis via regulating miR-942-5p-targeted PCP4, thus potentially preventing the progression of OP. This study for the first time explores the potential role of circ_0019693 in OP, which may help to develop new strategies for OP therapy.

## Highlight

- Circ_0019693 is downregulated in osteoporosis.
- Circ_0019693 overexpression promotes BMSC osteogenic differentiation and osteogenesis-coupled angiogenesis.
- Circ_0019693 competitively binds to miR-942-5p to regulate PCP4 expression.
- Circ_0019693 governs the miR-942-5p/PCP4 axis to suppress osteoporosis development.
